# Cetuximab-Immunoliposomes Loaded with TGF-β1 siRNA for the Targeting Therapy of NSCLC: Design, and In Vitro and In Vivo Evaluation

**DOI:** 10.3390/ijms26031196

**Published:** 2025-01-30

**Authors:** Yanan Shi, Houqian Zhang, Hao Chen, Jianwei Guo, Ranran Yuan, Yu Tian, Quanlin Xin, Zhen Mu, Yuping Tao, Yongchao Chu, Aiping Wang, Zhiwen Zhang, Jingwei Tian, Hongbo Wang

**Affiliations:** 1 Key Laboratory of Molecular Pharmacology and Drug Evaluation, Ministry of Education, School of Pharmacy, Ministry of Education, Collaborative Innovation Center of Advanced Drug Delivery System and Biotech Drugs in Universities of Shandong, Yantai University, Yantai 264005, China; zhanghouqian99@163.com (H.Z.); 15165536938@163.com (H.C.); 15239913157@163.com (R.Y.); 18769933062@163.com (Y.T.); xinquanlin813@163.com (Q.X.); muz990122@163.com (Z.M.); typ2016yd@163.com (Y.T.); ytuyongchao@163.com (Y.C.); wangaiping@luye.com (A.W.); tianjingwei618@163.com (J.T.); 2 Shandong Laboratory of Advanced Materials and Green Manufacturing at Yantai, Yantai 264006, China; jwguo@amgm.ac.cn; 3 School of Pharmacy, Fudan University, Shanghai 200437, China; zhangzhiwen@fudan.edu.cn

**Keywords:** non-small-cell lung cancer, TGF-β_1_ siRNA, gene silencing, immunoliposomes, antitumor

## Abstract

Transforming growth factor-β_1_ (TGF-β_1_) promotes the growth and metastasis of lung cancer cells. Therefore, TGF-β_1_ siRNA (siTGF-β_1_) gene therapy was introduced to inhibit the expression of TGF-β_1_ at the nucleic acid level to avert tumor growth and metastasis. However, the delivery of naked siRNA is typically restricted by a short half-life in vivo, difficulties in delivery in vivo, and safety issues. Using siTGF-β_1_ as a model drug, we established an actively targeted immunoliposome delivery system to investigate the role of siTGF-β_1_ in non-small-cell lung cancer (NSCLC). The results showed that the constructed immune liposomes were in a position to deliver siTGF-β_1_ to tumor cells, thus achieving a series of effects such as improving the poor stability and short half-life of naked siRNA. RNA interference of siTGF-β_1_ reduced the cell viability, growth, and migration potential of human non-small cell lung cancer cells (A549). Moreover, in an A549 tumor-bearing nude mouse model, siTGF-β_1_ transfection markedly reduced tumor growth and tumor volume. Inhibiting TGF-β_1_ diminished cancer cell viability and migration and promoted apoptosis in NSCLC, as confirmed by the findings of this study. Therefore, targeting siTGF-β_1_ with immunoliposomes may be a new therapeutic strategy for treating non-small-cell lung cancer.

## 1. Introduction

Non-small-cell lung cancer (NSCLC) is a common malignancy with high incidence [1] that is difficult to treat due to malignant migration and metastasis. In the past, the predominant lung cancer treatment avenues were chemotherapy and radiotherapy; however, traditional platinum-based chemotherapy schemes had limited benefits in terms of overall survival rates, and deteriorating physical conditions severely affected the quality of life of patients [2]. In 2004, mutations in the epidermal growth factor receptor (EGFR) were considered to be associated with NSCLC [3]. Approximately 10–30% of NSCLC neoplasms exhibit activating mutations in the tyrosine kinase structural domain of the EGFR gene, with an increased incidence of up to 60% in Asians patients [4]. In patients with metastable NSCLC with sensitized EGFR mutations, the first line of treatment is Osimertinib. Further first-line options approved by the FDA include Dacomitinib, Afatinib, Erlotinib, and Geftinib [5]. The two most common EGFR activation mutations are deletions in exon 19 and amino acid substitutions in exon 21, which make up 90% of the respective known mutations [6]. Therefore, EGFR mutations are biomarkers of lesions and therapy targets.

Transforming growth factor-β (TGF-β), particularly TGF-β1, is a key regulator of cell growth and is frequently upregulated in tumor cells, including those of non-small-cell lung cancer (NSCLC) [7]. TGF-β1 activation and Smad3 phosphorylation promote tumor growth and metastasis, highlighting the importance of targeting TGF-β1 for therapeutic interventions [7,8]. RNA interference using small interfering RNA (siRNA) offers a promising strategy to downregulate TGF-β1 expression. However, naked siRNA suffers from poor stability, a short half-life, and potential off-target effects, limiting its clinical application [9]. To address these challenges, various delivery systems have been developed, including viral vectors, which are highly efficient but limited by their toxicity and immunogenicity, and non-viral vectors such as cationic lipids and nanoparticles, which offer better biocompatibility and safety [10].

Regarding siRNA-loaded carriers, Polyethylene Glyco(PEG)-modified cationic liposomes are frequently used for siRNA transfection in vivo. PEG modification can protect siRNA liposomes from degradation by blood components such as macrophages, thus prolonging their retention time in blood circulation [11]. However, this approach exhibits negligible targeting effects. Tumor surface-associated antigens can be specifically bound by monoclonal antibodies, and subsequently, the drug or gene is translocated to the tumor cells with almost no effect on normal tissue. Cetuximab (CTX) is a monoclonal antibody chimeric with human/mouse derivative IgG1, which binds to the extracellular domain of EGFR with higher affinity than any endogenous ligand [12]. The use of CTX for NSCLC treatment has been widely proposed and has exhibited promising results [13,14,15]. Further, several studies have successfully linked CTX with nanocarriers to improve targeted delivery [15,16].

**Figure 1:**
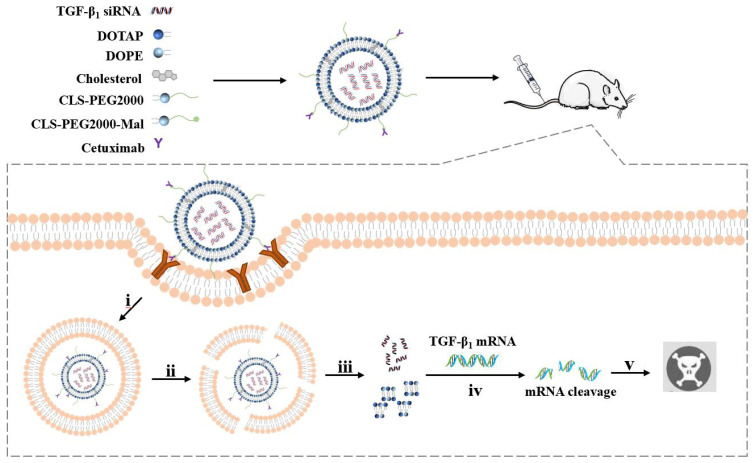
Schematic of the construction of immunoliposomes encapsulating siRNA, and intracellular transport of the delivery system, including endocytosis into endosomes (i), endosome escape (ii), liposome breakdown and siRNA release (iii), downregulation of TGF-β_1_ mRNA expression (iv), and RNAi-induced increase in apoptosis and decrease in proliferation (v).

In this study, PEG cationic liposomes were modified with CTX to load TGF-β1 siRNA (siTGF-β1) for delivery to the tumor site, where the siRNA exerts gene silencing and inhibits TGF-β1 expression at the nucleic acid level. A further objective was to determine that the knockdown of TGF-β_1_ can achieve the purpose of inhibiting tumor growth and metastasis (Figure 1). After the successful construction of immunoliposomes, we assessed cytotoxicity, cell uptake, lysosome escape, cell migration and invasion in A549 cells. We further conducted tissue distribution and pharmacodynamic experiments in A549 tumor-bearing nude mice and evaluated treatment safety. We propose synthesizing siRNA delivery platforms for active targeted delivery, thus inspiring novel strategies for the treatment of NSCLC.

## 2. Results

### 2.1. Characterization of Liposomes

**Figure 2:**
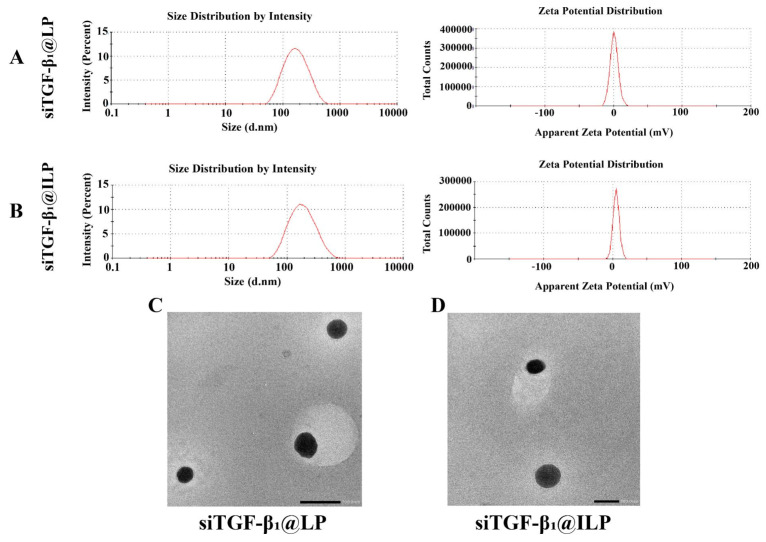
Transmission electron microscopy (TEM) images, particle size, and zeta potential of siTGF-β_1_@LP (**A**,**C**) and siTGF-β_1_@ILP (**B**,**D**) (*n* = 3).

PEGylated siTGF-β1 cationic liposomes (siTGF-β_1_@LP) was composed of DOTAP/DOPE/CH/CLS-PEG2000/CLS-PEG2000-Mal (molar ratio 1:3:1.5:0.12:0.03) and was then functionalized with cetuximab. Liposomes with CLS-PEG2000-Mal were used to couple thiolated cetuximab onto the distal end of the PEG polymer, forming immunoliposomes (siTGF-β1@ILP) with a connection efficiency of 62.02% ± 5.12%. DLS analysis showed that the average particle size of siTGF-β_1_@LP was 151.0 ± 1.3 nm, the polydispersity index (PDI) was 0.176 ± 0.018, and the potential was 1.2 ± 0.1 mV. After the functionalization of cetuximab, the average particle size of siTGF-β_1_@ILP increased to 159.2 ± 0.8 nm, the average value of PDI was 0.214 ± 0.012, and the potential was 4.9 ± 0.5 mV (Figure 2A,B). The morphology of siTGF-β_1_@LP and siTGF-β_1_@ILP as observed by TEM is shown in Figure 2C,D. After drug loading or functionalizing liposomes with cetuximab, siTGF-β_1_@LP and siTGF-β_1_@ILP retained their spherical shape and uniform particle size.

**Figure 3:**
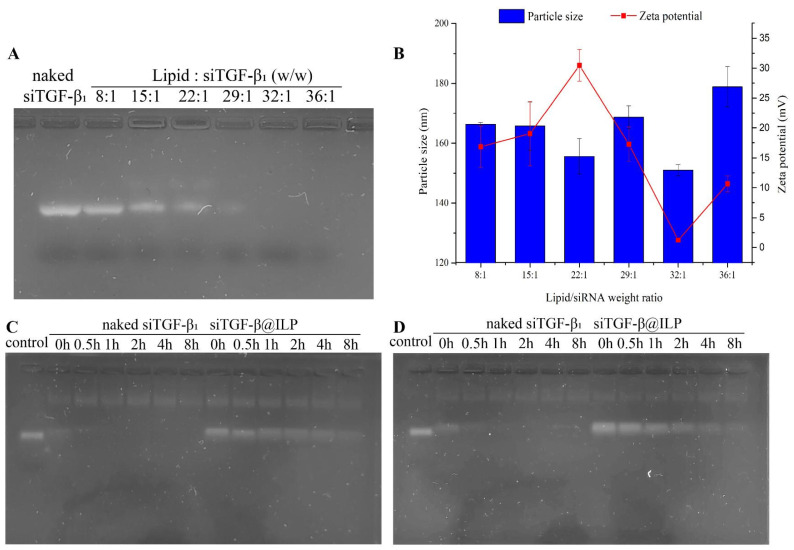
(**A**) Complexation of siRNA with siTGF-β_1_@ILP in agarose gel at various lipid/siRNA ratios (*n* = 3). (**B**) Particle sizes and zeta potentials of siTGF-β_1_@ILP formed at different lipid/siRNA ratios. Shown are the means ± SD (*n* = 3). Stability of naked siTGF-β_1_ and siTGF-β_1_@ILP after treatment with RNase (**C**) or serum (**D**) at different times (*n* = 3).

Agarose gel block tests showed that the liposomes and siTGF-β_1_ were completely complexed with a lipid:siTGF-β_1_ mass ratio of approximately 32:1 or higher, because all siRNA oligomers were loaded into liposomes to form high-molecular-weight complexes without any strip of free siRNA (Figure 3A). The relatively low weight ratio may offer the possibility of loading more drugs with reduced toxicity [17]. Therefore, the siRNA complex with a mass ratio of 32:1 was used for further experiments (Figure 3B).

Further, we examined the serum stability and enzyme stability of siTGF-β_1_@ILP. One of the obstacles to siRNA delivery is the lack of stability during in vivo transmission; thus, siRNA protection of siTGF-β_1_@ILP was determined through gel block tests. The band of bare siTGF-β_1_ disappeared completely after 1 h of incubation with Ribonuclease A (RNase A) or serum. However, siRNA bands in siTGF-β_1_@ILP remained detectable for up to 8 h (Figure 3C,D). The results showed that siTGF-β_1_@ILP has excellent stability, can resist nuclease attack, and remains stable in serum, which ensures the activity of siRNA in vivo and the ability to increase the amount of siRNA delivered to tumor sites, resulting in pronounced gene silencing effects.

### 2.2. The Primary Source of In Vitro Cytotoxicity Is siTGF-β1

**Figure 4:**
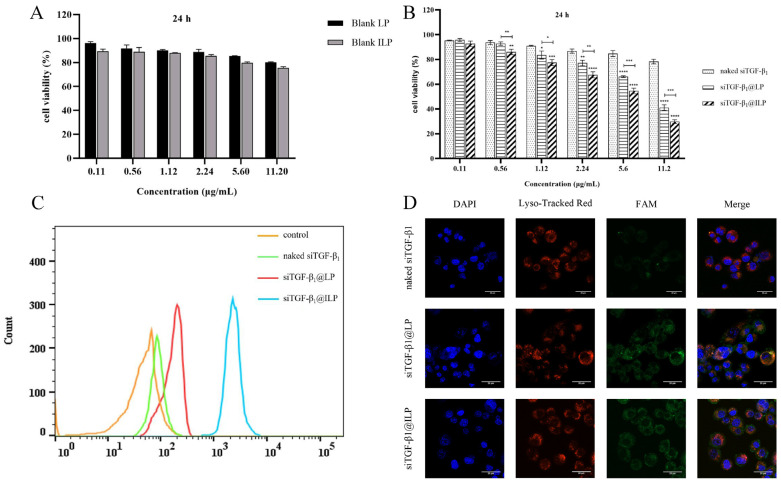
(**A**) In vitro cytotoxicity of blank LP and blank ILP at 24 h. Shown are the means ± SD (*n* = 3). (**B**) In vitro cytotoxicity of various liposomal formulations against A549 cells following 24 h incubation. Shown are the means ± SD (*n* = 3), * *p* < 0.05, ** *p* < 0.01, *** *p* < 0.001, **** *p* < 0.0001. (**C**) In vitro uptake study. Flow cytometric histogram profiles of fluorescence intensity of different formulations in A549 cells following 4 h of incubation at 37 °C (*n* = 3) are also shown. (**D**) Endosomal escape ability of siTGF-β_1_@LP and siTGF-β_1_@ILP in A549 cells. The blue fluorescence signal indicates the cell nucleus, red fluorescence indicates the stained lysosome, and the green fluorescence indicates siTGF-β_1_ labeled with FAM. Scale bar: 20 μm (*n* = 3).

An MTT assay was used to assess the cytotoxicity of naked siTGF-β_1_, siTGF-β_1_@LP, and siTGF-β_1_@ILP on A549 cells. As shown in Figure 4A,B, blank LP and blank ILP decreased cell viability in the range of 0.11 μg/mL to 11.20 μg/mL of siRNA concentration, because the high positive charge density of liposomes would indeed have increased cytotoxicity, and blank ILP also had a certain toxicity due to external CTX. In the same cell, all formulations, naked siTGF-β_1_, siTGF-β_1_@LP, and siTGF-β_1_@ILP, showed dose-dependent cytotoxicity, and their Half Maximal Inhibitory Concentration (IC_50_) values at 24 h were 274.4, 8.961, and 5.277 μg/mL, respectively. These results indicate that naked siRNA has no marked inhibitory effect on cells, which leads to the high survival rate of cancer cells during transfection. siTGF-β_1_@ILP showed stronger inhibition than siTGF-β_1_@LP, which confirmed that CTX attached to LP was active, enabling the functionalized liposome to have an active targeting effect, and was better able to restrain the growth and proliferation of cancer cells.

### 2.3. Cellular Uptake Study

To evaluate the uptake of siTGF-β_1_@LP and siTGF-β_1_@ILP by cells, flow cytometry was used for analysis after 4 h of treatment in the medium containing the drug, with free Fluorescein(FAM)-siRNA as a negative control. Flow cytometry showed a similar uptake of naked siRNA as in the blank control (Figure 4C). The uptake efficiency of the siTGF-β_1_@LP and siTGF-β_1_@ILP groups was higher than that of the naked siRNA group. These results were mainly because naked siRNA was degraded by intracellular enzymes without visible uptake, which also confirmed the protective effect of siTGF-β_1_@LP and siTGF-β_1_@ILP. When the cells were incubated with siTGF-β_1_@ILP, the concentration of FAM-positive A549 cells was markedly higher than when the cells were incubated with siTGF-β_1_@LP. The underlying reason is that in the presence of CTX modification, liposomes actively target tumor cells and internalize them, after which siRNA exerts gene silencing in the cytoplasm. This shows that the functionalized liposomes provide an ideal stage for siRNA entry into tumor cells.

### 2.4. DOPE and DOTAP: Enabling siTGF-β1 to Achieve Endosomal Escape

In the process of siRNA transfection, the internalized RNA is readily degraded by lysosome enzymes (such as acid RNA enzyme and exonuclease), resulting in reduced therapeutic efficacy [18]. Therefore, the endosome escape ability of siRNA complexes is crucial for siRNA to enter the cytoplasm to achieve gene silencing effects. DOPE has a pH responsive structural transition, which can destroy the stability of endosomal membrane in the acidic environment of endosomes, induce the fusion of liposome membrane and endosomal membrane, and promote drug release. DOTAP may also form ion pairs with endosome lipids, thereby disrupting the stability of the endosomes. Consequently, it is speculated that DOPE and DOTAP in the constructed liposomes ensure the endosomal escape of siRNA.

The escape ability from endosomes was tested using Confocal laser scanning microscope (CLSM). In the CLSM images (Figure 4D), the yellow signal on the overlapping area of red fluorescence (lysosomal red) and green fluorescence (siTGF-β_1_ labeled with green fluorescent FAM) indicates that siTGF-β_1_ was trapped within the endosome. After siTGF-β_1_ escaped from the endosome, bright green fluorescence was observed, and separation of green fluorescence and the red area was also detected. In the naked siTGF-β_1_ group, siTGF-β_1_ was degraded by intracellular enzymes at 4 h; thus, no substantial fluorescence occurred, and the small amount of fluorescence present was mainly in the endosome. In the siTGF-β_1_@LP and siTGF-β_1_@ILP groups, fast and effective endosome escape was observed even after 4 h, as pronounced green signals were observed, indicating that siRNA diffused from the endosome. Compared with the siTGF-β_1_@LP group, the red and green signals in the siTGF-β_1_@ILP group were separated more strongly. The siTGF-β_1_@LP group also showed some red/green fluorescence separation; however, it had more yellow signals. The results showed that the delivery system constructed could stably protect siRNA and promote the endosomes escape of siRNA, and the escape effect of the siTGF-β_1_@ILP group was stronger than that of the siTGF-β_1_@LP group. That is, when cetuximab was modified, liposomes could actively target tumor cells and escape from the endosomes, thus exerting the gene silencing effect.

### 2.5. Liposomes Inhibit the Invasion and Migration of A549 Cells

**Figure 5:**
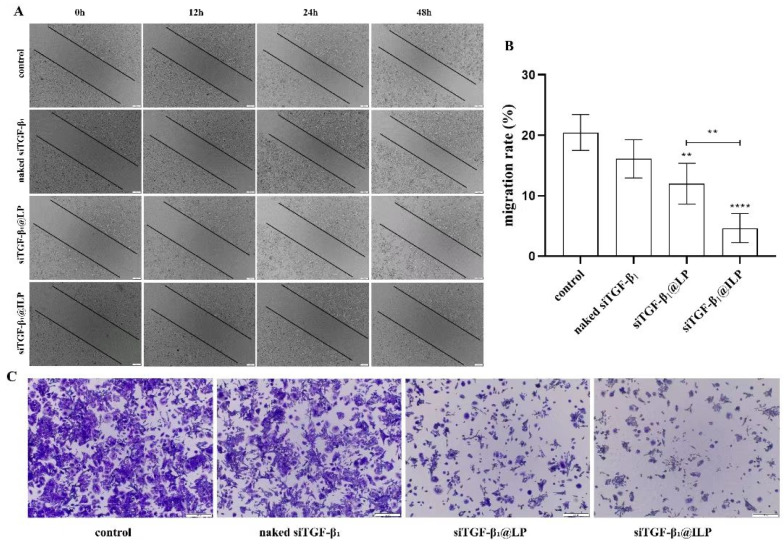
(**A**) Wound healing assay. Scale bar: 100 μm. (**B**) Migration rates of different complexes (*n* = 3) (** *p* < 0.01, **** *p* < 0.0001). (**C**) Transwell migration assay. Scale bar: 100 μm (*n* = 3).

The cell scratch test showed that the cell migration ability of the siTGF-β_1_@LP and siTGF-β_1_@ILP groups was lower than that of the naked siTGF-β_1_ group, and the cell migration ability of the siTGF-β_1_@ILP group was lower than that of the siTGF-β_1_@LP group (Figure 5A,B). In the transwell migration experiment, compared with the naked siTGF-β_1_ group, the amount of of cell invasion in the siTGF-β_1_@LP and siTGF-β_1_@ILP groups was markedly reduced (Figure 5C). These results confirm the successful delivery and efficient expression of siRNA in cells and show that the inhibition of TGF-β_1_ can effectively inhibit the growth and migration of A549 cells.

### 2.6. In Vivo Biodistribution of Liposomes in a Cell Line Xenograft Model

**Figure 6:**
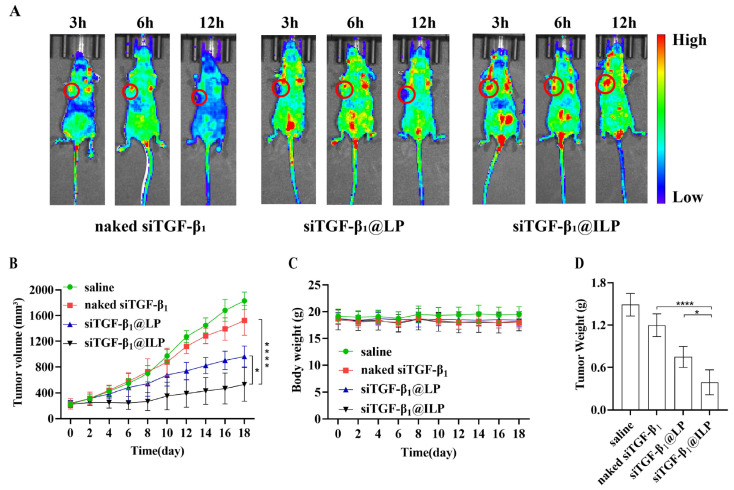
(**A**) In vivo distribution and imaging of siTGF-β_1_, siTGF-β_1_@LP, and siTGF-β_1_@ILP in tumors-bearing nude mice. (**B**) Tumor volume (mm^3^) of different groups. (**C**) Body weight changes of different groups. (**D**) Average tumor mass isolated from the mice of each experimental group. Shown are the means ± SD (*n* = 3–5); * *p* < 0.05, **** *p* < 0.0001.

Three hours after injection, free FAM-siRNA displayed faint fluorescence intensity at the tumor site, suggesting the instability and rapid clearance of naked siRNA in vivo, as shown in Figure 6A. By contrast, siTGF-β_1_@ILP containing the same amount of FAM-siRNA revealed distinct fluorescence at the tumor site, confirming stability in vivo and the successful delivery of siRNA to the tumor region via siTGF-β_1_@ILP, which was attributed to the active targeting and binding ability of CTX to EGFR. At the same time, siTGF-β_1_@ILP can prolong the complex circulation time (up to 12 h or even longer), thus increasing accumulation in the tumor. In the siTGF-β_1_@LP group, only a small fluorescence signal occurred at the tumor site, while a stronger signal intensity was observed in other parts of the body and was readily cleared by the reticuloendothelial system due to the positive charge.

### 2.7. In Vivo Anticancer Activity and Safety Evaluation

To assess the inhibition of tumor growth in vivo, an A549 tumor model was established using BALB/c nude mice. Various formulations, including free siTGF-β_1_, siTGF-β_1_@LP, and siTGF-β_1_@ILP, were injected through the tail vein once every 2 d. The normal saline group did not show inhibited tumor growth during the administration period and reached the maximum tumor volume (Figure 6B,D). The group administered free siRNA showed larger tumors at the end of treatment. The tumors in the siTGF-β_1_@LP group and siTGF-β_1_@ILP groups were smaller than those in the free siRNA- and saline-treated groups, whereas tumors in the siTGF-β_1_@ILP group were smaller than those in the siTGF-β_1_@LP group, with a substantial difference between them (*p* < 0.05). These data indicated that siTGF-β_1_@ILP has better selectivity and apparent growth inhibition effects on tumors, and siRNA embedded in siTGF-β_1_@ILP can achieve enhanced tumor inhibition and provide improved therapeutic effects, indicating that siTGF-β_1_@ILP may be a promising drug delivery system for NSCLC treatment.

**Figure 7:**
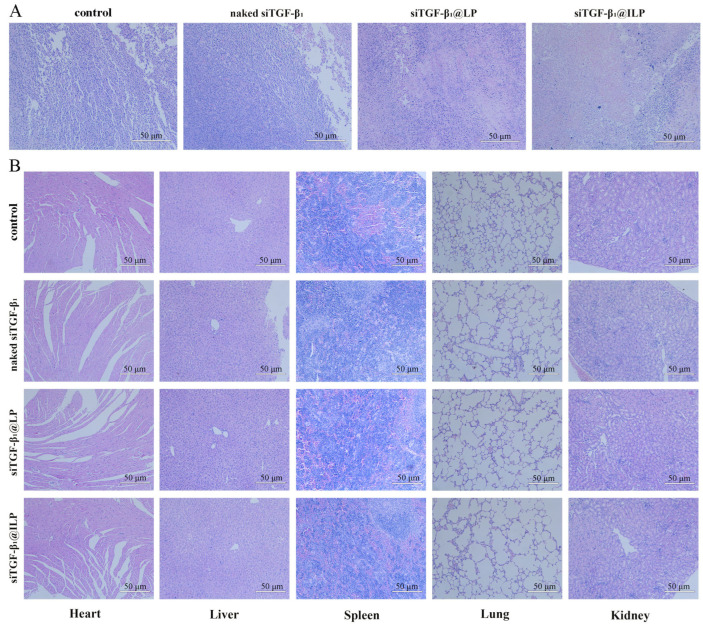
(**A**) Histological changes in tumor tissues after treatments, on day 18 (*n* = 3–5). (**B**) HE staining of major organs after treatment. Scale bar: 50 μm (*n* = 3–5).

Hematoxylin and Eosin staining (HE) showed histological changes in tumor tissues after treatment in each group (Figure 7A). The cytoplasm in normal tumor cells and in blood vessels was visible. After treatment with siTGF-β_1_@LP and siTGF-β_1_@ILP, tumor cell abundance decreased and the intercellular space increased, which was a typical characteristic of necrosis. The results showed that siTGF-β_1_@ILP was able to damage tumor tissue and inhibit tumor growth, and its effect was stronger than that of siTGF-β_1_@LP.

Considering that the preparation may cause immune reactions and systemic toxicity after intravenous injection, body weight changes in mice were recorded once every 2 d to evaluate the systemic toxicity of the vehicle in vivo (Figure 6C). None of the groups exhibited weight loss during the experiment, thus confirming the safety of siTGF-β_1_@LP and siTGF-β_1_@ILP. HE staining indicated that each group of preparations does not cause visible toxic pathological changes in the heart, liver, spleen, lung, or kidney (Figure 7B), which suggested the low toxicity of the constructed liposome.

### 2.8. Immunohistochemistry (IHC)

**Figure 8:**
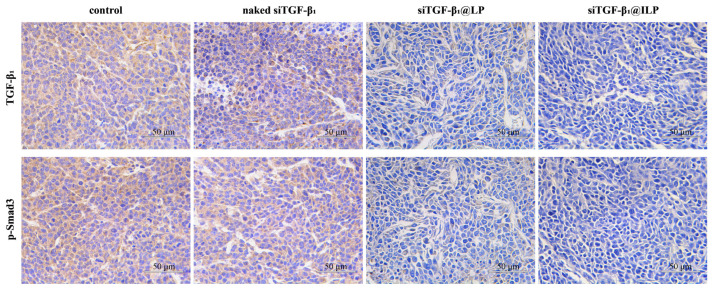
Immunohistochemistry (IHC) staining of tumor sections was used to detect the protein levels of TGF-β_1_ and p-Smad3. Scale bar: 50 μm (*n* = 3–5).

IHC results of tumor tissue showed no significant treatment effect in the siTGF-β1 group, whereas the siTGF-β_1_@ILP group showed stronger effects than the siTGF-β_1_@LP group. The expression levels of TGF-β_1_ and p-Smad3 were markedly reduced (Figure 8); thus, the growth and metastasis of A549 cells were prevented by inhibiting TGF-β/Smad signaling.

## 3. Discussion

Non-small-cell lung cancer (NSCLC) is the most common type of lung cancer, accounting for approximately 85% of all lung cancer cases [19]. As a malignant tumor originating in the lungs, NSCLC is characterized by high incidence and mortality rates and is often diagnosed at advanced stages, making it difficult to treat and resulting in poor prognosis [20]. TGF-β1 is a key member of the TGF-β superfamily and plays a complex and multifaceted role in the tumor microenvironment. TGF-β1 not only promotes the proliferation and survival of cancer cells but also induces epithelial–mesenchymal transition (EMT), thereby enhancing the invasiveness and metastatic potential of tumor cells. This makes TGF-β1 closely related to the development and progression of NSCLC [21,22,23]. Therefore, inhibiting the expression of TGF-β1 has become an important strategy for treating NSCLC.

siRNA technology, based on the principle of RNA interference (RNAi), is a powerful tool for gene silencing that can specifically degrade target mRNA, thereby inhibiting the expression of specific genes [24]. In recent years, siRNA technology has garnered significant attention for its potential applications in cancer therapy. Previous studies have shown that delivering siTGF-β1 via liposomes can effectively inhibit the expression of TGF-β1 in pulmonary epithelial cells and fibroblasts, thereby alleviating the symptoms of idiopathic pulmonary fibrosis (IPF) [25]. Additionally, research using bovine milk exosomes to deliver siTGF-β1 has demonstrated that this method can effectively transport siRNA to the lungs, inhibiting inflammatory responses, EMT, and the production of fibrosis-related molecules [26]. These studies provide a theoretical basis and practical references for utilizing siRNA technology to treat non-small-cell lung cancer (NSCLC).

Liposomes, as nanoscale vesicles composed of a phospholipid bilayer, are capable of encapsulating and protecting RNA molecules, ensuring their stability in the body and facilitating delivery to target cells [27]. However, traditional liposomes have several limitations, such as susceptibility to degradation and poor targeting ability. The positively charged character of DOTAP lipids allowed the negatively charged siRNA to easily compound with liposomes, and the cholesterol portion provided stability for the phospholipid bilayer, making it difficult for siRNA to escape from the liposome during delivery to the target sites [28,29,30]. During endocytosis, DOPE, a neutral auxiliary lipid, enables liposomes to destroy the endosomal membrane and release the loaded drug into the cytoplasm [29,31]. By contrast, grafting CLS-PEG2000 to the liposome surface was expected to potentially reduce blood circulation by avoiding cationic liposome aggregation and avoiding non-specific uptake in the reticuloendothelial system [32].

In this study, we selected PEGylated cationic liposomes as the carrier for siRNA and modified them with cetuximab to enhance their targeting ability and gene silencing activity. Cetuximab is a monoclonal antibody targeting the extracellular domain of the epidermal growth factor receptor (EGFR) and inhibiting its activation [14]. Overexpression of EGFR is common in NSCLC [33]; therefore, liposomes modified with cetuximab can specifically target tumor cells and improve the delivery efficiency of siRNA [15,34]. Experimental results showed that these modified liposomes could effectively bind siRNA and maintain stability in serum, protecting siRNA from RNase degradation.

Cytotoxicity assays revealed that naked siTGF-β1 had limited inhibitory effects on A549 cells, whereas siTGF-β1@LP and siTGF-β1@ILP, encapsulated in liposomes, significantly enhanced the inhibitory effects on A549 cells. Notably, cetuximab (CTX)-modified siTGF-β1@ILP exhibited stronger cytotoxicity, indicating that its active targeting ability enabled more effective siRNA delivery to tumor cells, thereby achieving better gene silencing effects. Wound healing assays further validated the inhibitory effects of siTGF-β1@LP and siTGF-β1@ILP on the migratory capacity of A549 cells, with siTGF-β1@ILP showing more pronounced effects. These results provide strong experimental evidence for TGF-β1 as a therapeutic target for non-small-cell lung cancer (NSCLC).

In in vivo experiments, siTGF-β1@ILP effectively delivered siRNA to tumor tissues through the targeting action of cetuximab, which is consistent with the stability of siRNA and cellular uptake observed in vitro. Pharmacodynamic tests demonstrated that siTGF-β1@ILP exhibited excellent activity in inhibiting tumor growth and did not cause significant systemic toxicity during the experimental period. Hematoxylin and eosin (HE) staining results showed that neither siTGF-β1@LP nor siTGF-β1@ILP induced apparent toxic pathological changes in major organs, confirming their good biocompatibility. In patients with non-small-cell lung cancer (NSCLC), plasma levels of TGF-β are significantly elevated compared to individuals without lung cancer, and this elevation is closely associated with low survival rates [35]. TGF-β1 significantly promotes the proliferation, migration, and invasion of tumor cells by activating the Smad3 signaling pathway [36]. IHC results indicated that siTGF-β1@ILP, modified with cetuximab, achieved active targeting, significantly enhanced the enrichment and gene silencing effects of siRNA in tumor cells, and exhibited strong inhibitory effects on TGF-β1 and p-Smad3 protein expression. The reduced expression levels of TGF-β1 and p-Smad3 proteins further confirmed that siTGF-β1@ILP effectively inhibited tumor cell proliferation and migration by suppressing the TGF-β/Smad signaling pathway, thereby exerting significant antitumor effects. Overall, as a targeted delivery system, siTGF-β1@ILP demonstrated excellent antitumor activity and safety in vivo and holds potential as a therapeutic strategy for non-small-cell lung cancer (NSCLC).

Despite the positive progress achieved in this study, there is still room for further optimization. First, TGF-β1 is only one factor in the TGF-β signaling pathway, and whether other members of the TGF-β family also have potential therapeutic effects on NSCLC requires further validation. Second, the low drug loading capacity of siRNA has always been a challenge for this technology, and how to increase the drug loading and delivery efficiency of liposomes will be a key focus for future research. In summary, by encapsulating siTGF-β1 in liposomes and modifying them with PEG and cetuximab (CTX), we successfully developed a targeted delivery system that effectively inhibits the expression of TGF-β1. This system not only demonstrated excellent antitumor activity and biocompatibility in both in vitro and in vivo experiments but also provided new insights and strategies for the treatment of NSCLC.

## 4. Materials and Methods

### 4.1. Cell Culture and Materials

A549 cells were acquired from Luye Pharmaceutical Co., Ltd. (Yantai, China) and were incubated in DMEM medium (Procell Life Science & Technology Co., Ltd., Wuhan, China) with 10% (*v*/*v*) FBS, and 1% (*v*/*v*) penicillin–streptomycin. The cells were maintained at 37 °C in a humidified atmosphere containing 5% CO_2_. FBS was obtained from Procell Life Science & Technology Co., Ltd. (Wuhan, China).

(2,3-Dioleoyloxy-propyl)-trimethylammonium-chloride (DOTAP), 1,2-dioleoyl-sn-glycero-3-phosphoethanolamine (DOPE), cholesterol (CH), Cholesterol-PEG2000 (CLS-PEG2000), and Cholesterol polyethylene glycol maleimide (CLS-PEG2000-Mal) were acquired from RVT Pharmaceutical Technology Co., Ltd. (Shanghai, China). Cetuximab was sourced from Weisman Bioengineering Co., Ltd. (Wuhan, China). All siRNA oligos including FAM-labeled siRNA were acquired from Gene Pharma (Shanghai, China), and the sense and antisense sequences of siTGF-β_1_ were *5′-GCAACAACGCCAUCUAUGATT-3′* and *5′-UCAUAGAUGGCGUUGUUGCTT-3′*, respectively. All other chemicals were of analytical or chromatographic grade.

### 4.2. Preparation of Liposomes

siTGF-β_1_ cationic liposomes were prepared using a modified ethanol injection method [37]. The lipid mixture composed of DOTAP, DOPE, and CH (molar ratio 1:3:1.5) was dissolved in an appropriate amount of absolute ethanol; the product was then transferred it to a glass bottle which was immediately placed in a water bath at 65 °C, and the liquid was stirred until the ethanol was volatilized. To load siTGF-β_1_, we added 5 mL diethyl pyrocarbonate water containing 280 μg siTGF-β_1_ to hydrate the dry lipid membrane. We used an ultrasonic cell disruptor for treatment in an ice water bath with 150 W for 15 min. The resulting siTGF-β_1_ liposomes were consecutively passed through microporous filter membranes of a 0.80, 0.45, and 0.22 µm mesh. Finally, polyethylene glycol liposomes were prepared using a post-insertion method, and CLS-PEG2000 and CLS-PEG2000-Mal (molar ratio 0.12:0.03) were slowly added to the liposomes [32].

After siTGF-β_1_@LP were prepared, cetuximab was used to functionalize the liposomes to obtain siTGF-β_1_@ILP [38]. Cetuximab and Traut’s reagent were sulfhydrylated at a molar ratio of 1:20 [15]. Thiolated cetuximab was incubated with the liposomes for 20 h at room temperature in the dark to bind the thiolated antibody to the liposome. Unbound cetuximab was removed by ultrafiltration tube centrifugation (molecular weight cut-off (MWCO) = 300 K; Amicon, Burlington, MA, USA), followed by disinfection by filtration through a sterile filter (0.22 µm) to obtain siTGF-β_1_@ILP. The product was stored at 4 °C. The conjugation efficacy of antibodies on liposomes was evaluated with the following equation: (M_t_ − M_u_)/M_t_ (M_t_: the mass of total antibodies; M_u_ represents the mass of unconjugated antibodies, and the antibodies were measured by the BCA Protein Assay Kit). The process for the preparation of FAM-siRNA-loaded liposomes was identical to that of siTGF-β_1_ liposomes.

### 4.3. Physicochemical Characterization of Liposomes

#### 4.3.1. Size and Zeta Potential Measurements

The particle size and zeta potential of siTGF-β_1_@LP and siTGF-β_1_@ILP were analyzed using a Malvern Instrument (Zetasizer Nano ZS, Malvern, UK). The morphology of siTGF-β_1_@LP and siTGF-β_1_@ILP was assessed using transmission electron microscopy (TEM; JEM-1400 Plus, Tokyo, Japan). Liposomes were negatively stained with a droplet of 2% phosphotungstic acid negative staining solution. Data were collected at an accelerating voltage of 80 kV.

#### 4.3.2. Gel Electrophoresis

Gel-blocking assays were used to detect the formation of siRNA–liposome complexes [39]. The complexes were prepared with different siRNA concentrations, and samples with different mass ratios (10 μL) were analyzed through 1.5% agarose gel electrophoresis (100 V, 30 min). Then, the gel was stained with Fluorescent Deoxyribonucleic Acid (DNA) loading buffer, and the stained bands were visualized under ultraviolet light.

#### 4.3.3. siRNA Protection

For assessing the serum stability and protection of siRNA in RNAase, free siTGF-β_1_ or equivalent siTGF-β_1_@ILP was mixed with equal volumes of FBS (50% serum concentration) or RNase A (1 mg/mL) at 37 °C. Samples collected at various time points were stored at −80 °C. Before gel electrophoresis, 10% heparin sodium solution was added to the samples to extract siRNA; gel electrophoresis was performed as described above. Untreated free siRNA was used as a control.

### 4.4. In Vitro Cytotoxicity

The cytotoxicity of naked siTGF-β_1_, siTGF-β_1_@LP, and siTGF-β_1_@ILP (*n* = 3, each) was assessed using an MTT assay [40]. A549 cells were trypsinized and were seeded into 96-well plates at 5 × 10^3^ cells per well. After 24 h, different concentrations of naked siTGF-β_1_ and siTGF-β_1_ loaded liposomes (equivalent to an siTGF-β_1_ of 0.11, 0.56, 1.12, 5.6, and 11.2 µg/mL) were spiked into each well of treated cells. Then, the cells were cultured for 24 h. Thereafter, 20 μL of MTT solution (5 mg/mL) was added to each well, followed by incubation for 4 h. The supernatant was removed, 200 μL of dimethyl sulfoxide was added, and the culture was shaken for 10 min to dissolve formazan crystals. A microplate reader (Model 550, Bio-Rad, Hercules, CA, USA) was used to assess absorbance at 570 nm for each well, and the untreated group acted as a control to calculate cell viability. Blank liposomes and blank immunoliposome were also evaluated using the same protocol.

### 4.5. Cellular Uptake

A549 cells were inoculated in six-well plates at a density of 2 × 10^5^ cells per well and were cultivated at 37 °C overnight for cell uptake analysis. The original substrate was then substituted with new DMEM containing naked siTGF-β1, siTGF-β1@LP, and siTGF-β1@ILP (siTGF-β_1_ labeled with FAM). The ultimate concentration of FAM-labeled siTGF-β_1_ was 100 nM [41]. After incubation at 37 °C for 4 h, the cells were washed using PBS. For the quantification of cellular uptake, cells were trypsinized, centrifuged, and resuspended in 200 µL PBS, after which they were assayed for fluorescence intensity using a flow cytometer (BD Biosciences, San Jose, CA, USA). The experiment was performed in triplicate (*n* = 3).

### 4.6. Endosomal Escape

A549 cells were plated at 2 × 10^5^ cells/well in a six-well plate and were placed in an incubator at 37 °C overnight. Then, the medium was replaced with 2 mL new medium containing naked siTGF-β1, siTGF-β1@LP, and siTGF-β1@ILP (siTGF-β_1_ labeled with FAM). The medium containing the complex was aspirated after 4 h of culture, and cells were washed twice with PBS and were then incubated with a lysosomal red fluorescent probe at 37 °C for 1 h to stain the lysosomes. The cells were then immobilized with 4% paraformaldehyde, and nuclei were 4′,6-Diamidino-2-phenylindole-stained (DAPI-stained) for 30 min, followed by examination and photography using CLSM. The experiment was performed in triplicate (*n* = 3).

### 4.7. Cell Migration Assay

#### 4.7.1. Wound Healing Assay

A549 cells were cultured in six-well plates at a density of 2 × 10^5^ cells for 24 h to achieve 90% monolayer fusion. A sterile pipette tip (200 μL volume) was then used to scratch the monolayer, followed by washing twice with PBS [42]. The cells were treated with naked siTGF-β1, siTGF-β1@LP, and siTGF-β1@ILP, and were cultured in DMEM containing 10% FBS for 48 h. The untreated group was used as a control. Wound width was measured using an inverted microscope (TS2R-FL, Tokyo, Japan) at 0, 12, 24, and 48 h. The cell migration rate was calculated as follows: (0 h scratch width − 48 h scratch width)/0 h scratch width × 100%. The experiment was performed in triplicate (*n* = 3).

#### 4.7.2. Transwell Migration Assay

For the transwell migration test, A549 cells were resuspended in serum-free medium at a concentration of 3 × 10^4^ cells/mL and were inoculated onto the upper surface of the transwell cavity [43]. Then, 10% FBS medium containing naked siTGF-β1, siTGF-β1@LP, and siTGF-β1@ILP (*n* = 3, each) was placed on the bottom of the chamber. After 24 h of cultivation, cells invading the lower surface of the chamber were immobilized with 4% paraformaldehyde and were stained using crystal violet solution. Then, the stained cells were washed using PBS and were recorded using an inverted microscope.

### 4.8. In Vivo Biodistribution of Liposomes in the Cell Line Xenograft Model

The A549 cell suspension (2 × 10^6^ cells/100 μL) was subcutaneously injected into the flanks of BALB/c nude mice (five weeks old, male, 18–20 g). After tumor volumes reached approximately 200 mm^3^, the A549 tumor-bearing mice were injected via the tail vein with naked FAM-siRNA or FAM-siRNA@LP/FAM-siRNA@ILP (siRNA concentration was 100 nM, 100 μL). Whole-body fluorescence images in fluorescence channels of FAM were measured 3, 6, and 12 h after injection using IVIS spectroscopy (Caliper Life Science, Hopkinton, MA, USA) and IVIS in vivo imaging software (Version 4.4). The measurement was conducted in triplicate (*n* = 3).

### 4.9. In Vivo Antitumor Effect and Biosafety

When the xenografted tumors grew to approximately 200 mm^3^, the nude mice were randomly assigned to four groups (*n* = 5, each), which were treated with saline, naked siTGF-β_1_, siTGF-β_1_@LP, or siTGF-β_1_@ILP via tail vein injection (100 nM, 100 μL) every 2nd day until day 18. Tumor volumes were measured to monitor the treatment effects in each group. Furthermore, the weights of A549 tumor-bearing mice were recorded to evaluate the systemic toxicity of the tested complexes. The mice were killed on day 18, and tumors and major organs including the heart, liver, spleen, lungs, and kidneys were harvested. The organ tissue was embedded in paraffin and was sliced into 5 μm thick sections for HE staining. A light microscope was employed to capture images to examine the anti-tumor effects and in vivo safety of the various siRNA preparations. The experiment was performed in triplicate (*n* = 3–5).

### 4.10. IHC of Tumor Tissue

The resected tumor tissue was fixed with 4% paraformaldehyde, embedded and sectioned with conventional paraffin, dewaxed, rehydrated, repaired with heat-induced epitopes, incubated with the respective primary antibodies at 4 °C overnight, and was dripped with the secondary antibody at room temperature for 1 h. Diaminobenzidine coloration, hematoxylin double-dyeing, and dehydration were performed. The staining results were observed using a light microscope. The experiment was performed in triplicate (*n* = 3–5).

### 4.11. Statistical Analyses

The data are shown as the means ± standard deviation (SD) from independent experiments. Differences between groups in continuous variables were analyzed using Student’s *t*-test. Differences between multiple groups were analyzed using a one-way ANOVA. Statistical significance is reported at *p* < 0.05.

## 5. Conclusions

In conclusion, these assays strongly suggest that the prepared siTGF-β_1_@ILP based on cetuximab-functionalization was an excellent system to deliver TGF-β_1_ siRNA to A549 cells, which could enhance the gene silencing effect of siRNA by promoting tumor-targeting effects, so as to achieve the purpose of treating NSCLC. After functionalization with cetuximab, the delivery system effectively delivered siRNA to A549 cells in vitro and successfully directed the delivery system to tumor sites in vivo. Our results thus show a new drug delivery system with visible gene delivery capability, and the delivery of TGF-β_1_ siRNA by immunoliposomes will be a promising therapeutic method for NSCLC.

## Data Availability

The original contributions presented in this study are included in the article. Further inquiries can be directed to the corresponding authors.
